# A case report series of influenza A virus-induced pulmonary disease with diverse clinical manifestations

**DOI:** 10.3389/fmed.2026.1905517

**Published:** 2026-09-14

**Authors:** Linjie Guo, Yiwei Zhang, Xingguo Niu, Dalong Zhang

**Affiliations:** The Fifth Clinical Medical College of Henan University of Chinese Medicine, Zhengzhou, Henan, China

**Keywords:** airway hyperresponsiveness, case report, influenza A, severe pneumonia, VV-ECMO

## Abstract

**Background:**

Influenza A virus (IAV) typically causes influenza-like symptoms; however, in patients with underlying chronic respiratory diseases, it may progress to severe pneumonia and acute respiratory distress syndrome (ARDS). In addition to parenchymal lung injury, some patients may develop severe airway hyperresponsiveness (AHR), including bronchospasm, mucus plug formation, and refractory carbon dioxide retention, often resulting in poor responses to conventional respiratory support.

**Methods:**

This study reports three cases of IAV-associated pulmonary disease complicated by respiratory failure. Two patients were elderly men with COPD who presented with refractory AHR and carbon dioxide retention, despite the absence of severe pulmonary infection on imaging. The third patient was a middle-aged man without underlying disease whose chest imaging demonstrated severe pneumonia. All three patients received comprehensive treatment centered on respiratory support, including high-flow oxygen therapy, invasive mechanical ventilation, and individualized antimicrobial therapy; one patient additionally received veno-venous extracorporeal membrane oxygenation (VV-ECMO) support.

**Results:**

Despite aggressive treatment, including VV-ECMO support in one patient, the two patients with COPD experienced unfavorable outcomes. Case 1 developed persistent refractory AHR and progressive ventilatory failure, while Case 3 deteriorated in the context of multiple complications. In contrast, the patient without underlying disease, despite severe pneumonia on chest CT, recovered and was discharged following treatment with high-flow nasal oxygen therapy alone.

**Conclusion:**

In this case series, the clinical outcomes of IAV-associated pulmonary disease appeared to differ according to underlying pulmonary conditions. Patients with COPD exhibited more persistent AHR and ventilatory failure, whereas the patient without chronic respiratory disease showed reversible airway dysfunction and favorable recovery. These observations suggest that underlying respiratory status may influence the clinical course of IAV-associated AHR; however, further studies are required to validate these findings.

## 1 Introduction

IAV is a major respiratory pathogen that damages airway and alveolar epithelial cells (1) and triggers excessive inflammation, causing diffuse lung injury, pulmonary edema, impaired gas exchange, and progression to severe pneumonia or acute respiratory distress syndrome (ARDS).

In severe pneumonia, viral infection and inflammation (2) may induce airway hyperresponsiveness (AHR) through airway narrowing and mucus plugging, causing severe ventilatory failure, refractory hypercapnia (PaCO_2_ > 60 mmHg), and resistance to mechanical ventilation.

COPD is characterized by persistent airflow limitation (3, 4), chronic airway inflammation, emphysema, and mucus hypersecretion, an independent risk factor for exacerbation and mortality (5, 6). When IAV-induced AHR overlaps with COPD, ventilatory failure and CO₂ retention may worsen, while severe airway obstruction reduces ventilatory efficiency, necessitates higher airway pressures, and increases risks of barotrauma and hemodynamic compromise.

Although ECMO can support refractory respiratory failure, its optimal use and bleeding risks remain challenging. By presenting three patients with IAV-associated pulmonary disease showing different clinical phenotypes and outcomes, this case series aims to characterize severe AHR and explore the potential influence of underlying pulmonary conditions on treatment response and prognosis. These observations may provide a basis for further investigation of individualized management strategies.

## 2 Case reports

Currently, no universally accepted diagnostic criteria exist for influenza A virus-associated airway hyperreactivity (AHR). Therefore, in this case series, AHR was defined using a pragmatic clinical framework developed by the authors based on reported characteristics of severe airway hyperresponsiveness and clinical manifestations in critically ill patients. AHR was diagnosed according to the following clinical criteria:

1. clinical manifestations, including refractory prolonged expiration, wheezing, and poor responsiveness to bronchodilator therapy;
2. abnormal respiratory mechanics, characterized by markedly elevated peak airway pressure, reduced dynamic lung compliance, and difficulty maintaining adequate tidal volume;
3. arterial blood gas findings, particularly persistent hypercapnia (PaCO_2_ > 60 mmHg) despite ventilatory support;
4. bronchoscopic findings demonstrating airway mucosal congestion and edema, significant airway narrowing, and mucus plug formation;
5. exclusion of mechanical airway obstruction, such as airway secretions or endotracheal tube-related complications. These criteria were integrated to facilitate dynamic bedside assessment of both the presence and severity of AHR (Figure 1). The clinical characteristics and outcomes of the three patients are summarized in (Table 1).

**Figure 1:**
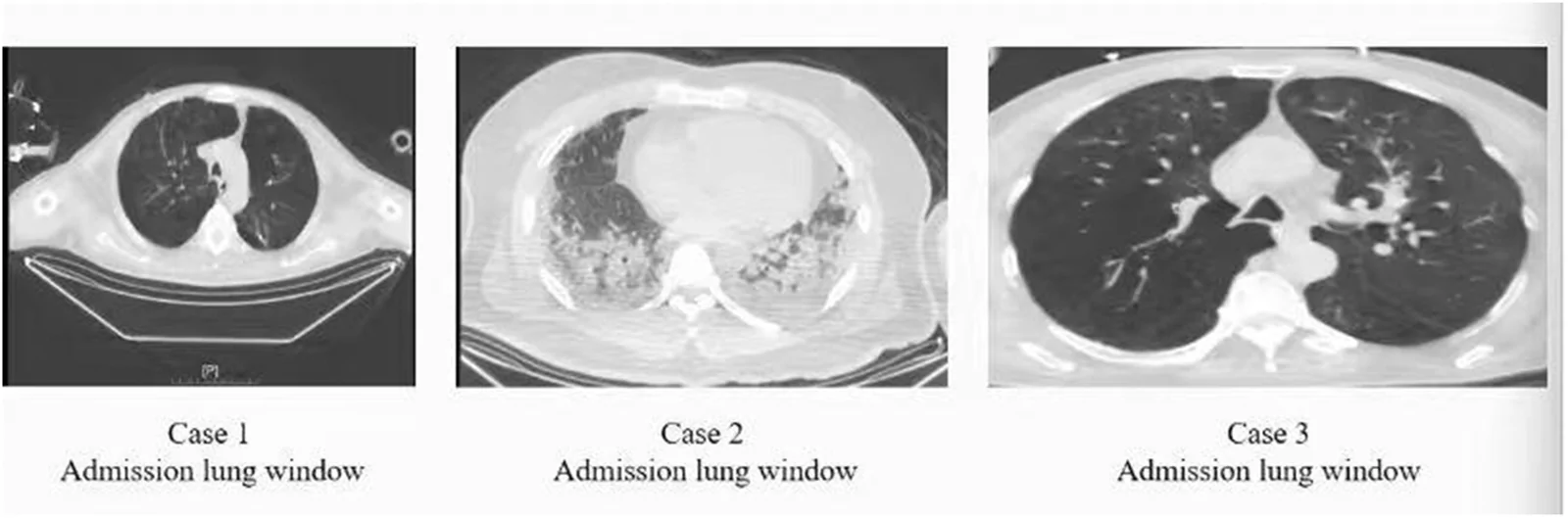
Admission lung window findings of the three cases.

**Table 1:** Clinical characteristics and outcomes of three patients with influenza A-associated pulmonary disease.

**Characteristics**	**Case 1**	**Case 2**	**Case 3**
Age/Sex	62/M	68/M	45/M
Underlying disease	COPD	COPD	None
Influenza A subtype	H1N1	H1N1	H3N2
AHR phenotype	Yes	Yes	Yes
PaCO_2_ elevation	Yes	Yes	No
Bronchoscopy evidence	Yes	No	No
Mechanical ventilation	Yes	Yes	Yes
VV-ECMO	No	No	Yes
Outcome	Death	Death	Survival

### 2.1 Case 1: elderly patient with COPD and influenza A

#### 2.1.1 Patient information

A 62-year-old man was admitted for dyspnea for 4 days, worsening over the preceding hour. He had COPD for >30 years, underwent pulmonary bullectomy 6 years earlier, and had received continuous home oxygen therapy via a personal oxygen concentrator for 2 years. He denied hypertension or diabetes mellitus and reported sulfonamide allergy. Four days before admission, while hospitalized elsewhere, worsening dyspnea and altered mental status required emergency endotracheal intubation and mechanical ventilation; influenza A testing was positive. Despite antiviral and antimicrobial therapy, inflammatory markers continued to rise, with hepatic dysfunction and blood pressure fluctuations, prompting transfer to our hospital for further management.

#### 2.1.2 Clinical findings

- On admission, the patient's temperature was 37 °C, pulse rate was 93 beats/min, and blood pressure was 106/74 mmHg. He remained under sedation. Bilateral pupils were equal in size, approximately 2.0 mm in diameter, with absent light reflexes{Neurological examination revealed absent pupillary light reflex during deep sedation [Richmond Agitation-Sedation Scale (RASS) −5]. Therefore, this finding was considered difficult to interpret as an independent neurological event.}. Coarse breath sounds were auscultated bilaterally, with wet rales audible in both lower lung fields.
- Respiratory monitoring demonstrated persistently elevated peak airway pressures (>35 cmH_2_O) accompanied by a reduction in tidal volume.
- Laboratory testing demonstrated markedly elevated inflammatory markers, including C-reactive protein (CRP) of 183.83 mg/L and procalcitonin (PCT) of 1.24 ng/mL, whereas the oxygenation index was 350. During AHR episodes, PaCO_2_ remained elevated, peaking at 80.4 mmHg, with increased peak airway pressure and decreased effective tidal volume. Adequate analgesia and sedation alleviated airway spasm and resistance and improved respiratory mechanics. Oxygenation was not significantly impaired, and low-concentration oxygen maintained stable oxygenation. Thus, the main abnormalities were AHR-related ventilatory dysfunction and CO_2_ retention. Changes in sedation and analgesia produced corresponding fluctuations in AHR and CO_2_ retention.
- The preliminary diagnoses were influenza A infection and acute exacerbation of chronic obstructive pulmonary disease (AECOPD). Hepatic dysfunction was also present, with alanine aminotransferase (ALT) of 468 U/L and aspartate aminotransferase (AST) of 275 U/L. This may be related to infection-associated systemic inflammation and reduced hepatic perfusion. Severe infection, inflammation, and hemodynamic instability may reduce hepatic perfusion and oxygen supply, causing hypoxic/ischemic hepatocellular injury, while inflammation-mediated liver injury cannot be excluded. Thus, the abnormal liver function was likely multifactorial, primarily involving systemic inflammation and impaired hepatic oxygenation.
- Chest CT demonstrated bilateral emphysema, scattered pulmonary bullae, multiple solid nodules, organizing changes, and localized bilateral pleural thickening.

#### 2.1.3 Interventions

- Respiratory support: The patient was intubated and mechanically ventilated, requiring only low-concentration oxygen to maintain good oxygenation. However, influenza virus-induced AHR caused marked airway resistance and expiratory limitation, resulting in refractory CO_2_ retention. Adequate analgesia and sedation were administered to reduce airway reactivity and improve ventilator–patient synchrony. Deep sedation partially relieved airway spasm and controlled airway pressure and CO_2_ levels, whereas tapering sedation and analgesia worsened AHR, with elevated airway pressure, increased dyspnea, and progressive CO_2_ retention. Persistent high airway pressure and abnormal respiratory mechanics prompted multiple fiberoptic bronchoscopies, revealing marked airway mucosal congestion and erythema. Regular glucocorticoids (dexamethasone) were administered to suppress airway inflammation and reduce AHR, while low-concentration oxygen ventilation was maintained continuously.
- Antimicrobial therapy: Empirical antimicrobial treatment with imipenem, teicoplanin, and oseltamivir was initiated. The empirical use of teicoplanin was primarily based on its antimicrobial activity against methicillin-resistant Staphylococcus aureus (MRSA). In addition, its relatively low nephrotoxicity makes it a more suitable option for critically ill patients who are at increased risk of multi-organ dysfunction.
- Liver function and nutritional support: Ammonia-lowering and hepatoprotective therapy was administered. Due to unstable vital signs, enteral nutrition was withheld and replaced with intravenous nutritional support, with close electrolyte and liver function monitoring.
- Coagulation management: Deep venous thrombosis involving the bilateral superficial femoral, popliteal, posterior tibial, peroneal, and intermuscular veins was identified during hospitalization. Low-molecular-weight heparin anticoagulation was initiated, and inferior vena cava filter placement was planned.
- Adjuvant therapy: Because of abdominal distension and impaired gastrointestinal function, adjunctive traditional Chinese medicine therapies were administered, including herbal poultices, acupoint plaster application, and moxibustion.

#### 2.1.4 Treatment outcomes and prognosis

The patient's AHR fluctuated dynamically thereafter, with refractory AHR persisting despite reduced analgesia and sedation. CO_2_ retention continued, with progressively increasing PaCO_2_, indicating severe ventilatory dysfunction. The ventilator simultaneously alarmed for excessive airway pressure and insufficient tidal volume. On January 13, 2025, refractory low tidal volume developed, followed by sudden bradycardia and hypotension unresponsive to vasoactive agents. The patient's family refused VV-ECMO. Despite aggressive resuscitation, vital signs could not be sustained, and the patient was pronounced dead.

### 2.2 Case 2: middle-aged patient without underlying lung disease

#### 2.2.1 Patient information

A 41-year-old previously healthy man was admitted with fever for 20 days, worsening over 7 days, and dyspnea for 3 h. He initially developed low-grade fever, followed 7 days before admission by fever up to 40 °C and cough. Nucleic acid testing was positive for influenza A (H1N1), with multiple infections including Acinetobacter baumannii. Chest CT showed bilateral multiple ground-glass opacities (GGOs), and the oxygenation index was 145, suggesting viral pneumonia. Arterial blood gases showed varying CO_2_ retention (PaCO_2_ up to 48.5 mmHg), suggesting ventilatory limitation from airway dysfunction in addition to impaired oxygenation from parenchymal injury. Three hours before admission, marked dyspnea developed, with SpO_2_ falling to 74%.

#### 2.2.2 Clinical findings

On admission, temperature was 37.5 °C, pulse 115 beats/min, and blood pressure 150/85 mmHg. Under high-flow oxygen therapy (FiO_2_ 50%), SpO_2_ was 93%. The patient was conscious and alert. Auscultation revealed bilateral coarse breath sounds with occasional moist rales and wheezing. CRP and PCT were elevated (55.94 mg/L and 0.373 ng/mL, respectively), and influenza A nucleic acid testing was positive. Lung ultrasound showed bilateral lower-lung B-lines.

#### 2.2.3 Interventions

- Respiratory support: Upon admission, high-flow nasal cannula (HFNC) oxygen therapy was immediately initiated because of hypoxemia, with a minimum peripheral oxygen saturation (SpO_2_) of 83%. The initial settings were a flow rate of 50 L/min and FiO_2_ of 50%, resulting in improvement of oxygen saturation to 95%. HFNC remained the primary mode of respiratory support throughout hospitalization. No endotracheal intubation, invasive mechanical ventilation, or noninvasive ventilation was required.
- Antimicrobial and immunomodulatory therapy: Antiviral treatment was sequentially administered with baloxavir marboxil and peramivir. Based on microbiological findings, including next-generation sequencing (NGS) of bronchoalveolar lavage fluid demonstrating mixed infection with Acinetobacter baumannii, Pseudomonas aeruginosa, and influenza A (H1N1) virus, antimicrobial therapy was progressively escalated to meropenem. Although the patient had no underlying immunosuppressive condition, relative immune dysfunction secondary to severe infection may have been present; therefore, thymalfasin and human immunoglobulin were administered as immunomodulatory therapy.The use of human immunoglobulin is primarily based on its potential immunomodulatory effects in severe pneumonia; however, there remains insufficient evidence to support its efficacy in the treatment of mild-to-moderate influenza infections.
- Fluid management and supportive care: Albumin infusion and diuretic therapy were administered. Fluid status was evaluated daily to maintain a negative fluid balance. Low-molecular-weight heparin was used for anticoagulation prophylaxis to prevent thrombosis.

#### 2.2.4 Treatment outcomes and course

The patient's dyspnea gradually improved, fever resolved, and inflammatory markers progressively decreased (CRP decreased from 55.94 mg/L to 1.40 mg/L, while PCT decreased to 0.040 ng/mL). As infection control measures took effect and airway inflammation improved, the patient's airway hyperresponsiveness gradually subsided. Arterial blood gas analysis showed that PaCO_2_ gradually returned to the normal range from a peak of 45.1 mmHg, indicating a concurrent improvement in ventilatory function. Oxygenation also improved continuously, and respiratory support was gradually downgraded from HFNC oxygen therapy (FiO_2_ 60%) to nasal cannula oxygen therapy. Eventually, the patient's condition stabilized completely, with resolution of cough and dyspnea, and he was discharged after full recovery.

### 2.3 Case 3: elderly patient with COPD requiring VV-ECMO

#### 2.3.1 Patient information

Patient Wu, a 66-year-old man, was admitted for dyspnea and altered mental status lasting >1 week. He had poorly controlled hypertension and diabetes mellitus for >10 years and COPD for >10 years. One week before admission, he developed dyspnea, fever (maximum 38.5 °C), and fatigue. A local hospital diagnosed influenza A infection, severe pneumonia, and respiratory failure; he received endotracheal intubation, mechanical ventilation, and antiviral therapy, but failed to improve.

#### 2.3.2 Clinical findings

On admission, temperature was 36.8 °C, pulse 81 beats/min, respiratory rate 25 breaths/min, and blood pressure 145/83 mmHg. The patient was mechanically ventilated via endotracheal intubation in the prone position, with bilateral diminished breath sounds and rapid respirations. Abdominal distension, faint bowel sounds, and mild bilateral lower-extremity edema were noted. Arterial blood gases over 7 consecutive days showed marked CO_2_ retention (PaCO_2_ 70–130 mmHg; pH 7.200). Blood pressure remained unstable, requiring high-dose sedation, analgesia, and vasopressors. Respiratory mechanics showed persistently elevated peak airway pressure (Ppeak 40–60 cmH_2_O) and plateau pressure (Pplat ∼30–40 cmH_2_O), indicating markedly increased airway resistance, with intrinsic PEEP (PEEPi ∼8–12 cmH_2_O) suggesting dynamic hyperinflation. Despite a set tidal volume of 450 mL, the delivered volume was only 150–220 mL, indicating severe ventilatory impairment.

#### 2.3.3 Interventions

- VV-ECMO support: Although guidelines recommend considering ECMO early in severe respiratory failure (generally within 7 days), the patient initially partially responded to high-intensity mechanical ventilation. Limited local resources and family reluctance to pursue ECMO led to progressive deterioration and increasingly difficult management; ECMO was initiated immediately after emergency transfer to a tertiary center. Conventional respiratory support, including increased pressure limits, high-dose sedation and analgesia, and neuromuscular blockade, failed to correct refractory hypercapnia. The patient developed suspected obstructive shock, with sudden bradycardia (40–50 beats/min) and difficulty maintaining blood pressure; VV-ECMO was therefore urgently initiated for CO₂ removal. An awake ECMO strategy was subsequently attempted and the endotracheal tube removed, but reintubation was required because of pulmonary hemorrhage. ECMO flow could not be gradually reduced, indicating persistent primary ventilatory failure.
- Antimicrobial therapy: Broad-spectrum antimicrobial therapy was administered throughout the clinical course. Early pathogen identification revealed influenza A virus, Enterococcus faecalis, Klebsiella pneumoniae, and Acinetobacter baumannii. Initial treatment consisted of imipenem-cilastatin combined with teicoplanin and oseltamivir. Later, due to progressive clinical deterioration complicated by multiple organ dysfunction syndrome and severe mixed infections, repeat pathogen testing identified carbapenem-resistant Klebsiella pneumoniae and carbapenem-resistant Acinetobacter baumannii, together with Aspergillus fumigatus, Aspergillus flavus, and human herpesvirus infection. In the later stage of illness, antimicrobial therapy was further adjusted to include ceftazidime-avibactam, eravacycline, polymyxin E (intravenous plus nebulized administration), isavuconazole, acyclovir, and caspofungin, among others. Eravacycline is approved for the treatment of complicated intra-abdominal infections (cIAI) in adults; therefore, its use in this case was off-label. The decision to administer this agent was primarily based on its documented *in vitro* activity against multidrug-resistant Acinetobacter baumannii, and it was selected as a salvage therapeutic option after other antimicrobial regimens demonstrated limited clinical efficacy.
- Management of airway hyperresponsiveness: Intravenous and nebulized corticosteroids, together with bronchodilators, were administered. Antimicrobial therapy was adjusted multiple times to control fungal and bacterial infections that might trigger airway reactions. To reduce elevated airway pressure, deep sedation and neuromuscular blockade were maintained under ECMO support.
- Management of complications: Diffuse intrapulmonary hemorrhage developed during treatment. Bleeding persisted despite discontinuation of anticoagulation with argatroban for 10 days during ECMO support. This complication was considered to be associated with infection, progressive disease deterioration, local procedures (including bronchoalveolar lavage and nebulization), and the patient's underlying conditions.

#### 2.3.4 Treatment outcomes and prognosis

Pulmonary imaging findings progressively worsened during ECMO support. Despite maximal treatment efforts, airway hyperresponsiveness, ventilatory failure, and pulmonary hemorrhage were never fundamentally controlled, and circulatory support eventually became unsustainable. Ultimately, the patient died of respiratory and circulatory failure.

## 3 Discussion

This case series reports three patients with severe influenza A pneumonia, in whom airway hyperresponsiveness was closely associated with ventilatory failure and prognosis. In Cases 1 and 3, COPD was accompanied by acute airway abnormalities, including bronchospasm, mucosal edema, and excessive mucus secretion, potentially causing airflow limitation and ventilatory failure. However, other factors, such as secondary infections, may also have influenced disease progression (7). Despite intensive respiratory support, both patients died. Although VV-ECMO can temporarily provide gas exchange support, it cannot correct the underlying airway obstruction or structural lung disease. In addition, the anticoagulation required during ECMO increases the risk of bleeding, particularly in patients with airway mucosal injury or inflammation (8). Short-term glucocorticoid therapy may reduce airway inflammation and mucosal damage, thereby improving airflow limitation with a relatively low bleeding risk; however, prolonged use may delay viral clearance and increase secondary infections (9).

The role of systemic corticosteroids in viral pneumonia remains controversial. Excessive immunosuppression may impair viral clearance and increase secondary infection risk, whereas corticosteroids may benefit severe AHR by suppressing airway inflammation, reducing bronchial mucosal edema, and improving airway responsiveness. In this series, corticosteroids targeted AHR-related airway inflammation, bronchospasm, and refractory hypercapnia rather than antiviral treatment. Thus, corticosteroid therapy should be individualized according to airway dysfunction severity, inflammatory status, and clinical progression rather than routinely used in all patients with influenza-associated pneumonia.

IAV can cause severe pneumonia and acute respiratory failure by directly damaging alveolar epithelial cells and inducing excessive airway inflammation, leading to AHR (AHR) (10). All three patients demonstrated clinical features compatible with this process. CRP and PCT were dynamically monitored to assess inflammatory activity, secondary infection, and treatment response. The primary clinical challenges facing these patients appear to be severe airflow obstruction and refractory hypercapnia associated with airway hyperresponsiveness, rather than hyperoxemia caused primarily by parenchymal lung injury (11).Cases 1 and 2 suggest that IAV-associated respiratory failure may have different clinical phenotypes. Unlike hypoxic respiratory failure from typical parenchymal injury, some patients may primarily develop AHR-related ventilatory dysfunction, characterized by increased airway resistance, expiratory limitation, and impaired CO₂ elimination. Thus, oxygenation indices such as PaO_2_/FiO_2_ alone may not adequately reflect respiratory dysfunction severity. In Cases 1 and 3, underlying COPD further worsened ventilatory impairment.

In contrast, Case 2 had no underlying lung disease. Despite severe IAV-induced AHR, the airway changes were reversible. With HFNC support and appropriate antiviral and antimicrobial therapy, the patient recovered completely and was discharged.

These three cases suggest that underlying chronic lung diseases (particularly chronic obstructive pulmonary disease) may be associated with more severe clinical manifestations and a poorer prognosis of influenza A-associated refractory airway hyperresponsiveness (AHR). In patients without pre-existing lung disease, AHR is an acute and reversible functional change, and outcomes are generally favorable. In contrast, patients with chronic obstructive pulmonary disease (COPD) in this series of studies developed persistent ventilatory failure, which may be related to the combined effects of acute airway inflammation and pre-existing structural abnormalities. Although one patient received VV-ECMO support, the limited number of cases precludes drawing any conclusions regarding its beneficial effect on survival.

These findings highlight the importance of early identification of underlying pulmonary disease and risk stratification for refractory AHR in severe influenza A pneumonia. Treatment should be individualized: patients without chronic lung disease may benefit from supportive care, including HFNC or non-invasive ventilation, whereas COPD patients require early recognition of severe ventilatory impairment and carefully adjusted ventilation to balance adequate ventilation and prevent injury.

In ECMO-supported patients, VV-ECMO primarily provides gas exchange support, with individualized anticoagulation to maintain circuit patency, but does not correct primary airway obstruction. Despite lung-protective ventilation (low tidal volume, limited plateau pressure, and prolonged expiratory time), severe AHR may cause persistent ventilatory failure. ECMO-associated bleeding, particularly pulmonary hemorrhage, remains a major complication requiring careful anticoagulation–bleeding risk balance.

Limitations of this study include a small sample size, lack of quantitative inflammatory marker analysis, and absence of long-term follow-up. Although no clear evidence suggested a dominant role of antiviral resistance, this cannot be fully excluded due to the lack of viral genetic testing and drug susceptibility analysis. In addition, all three patients included in this series were male, which may limit the generalizability of these findings to female patients. Clinically, Case 2 made a good recovery after receiving standard antiviral therapy and supportive care. In contrast, Cases 1 and 3 experienced worsening of their condition due to a combination of factors, including underlying chronic obstructive pulmonary disease (COPD) and secondary infections. Notably, Case 3 developed sequential late-stage mixed infections, suggesting that complex co-infections may contribute to disease progression.

In summary, this case series highlights that, in influenza A–associated AHR, underlying respiratory disease may represent an important factor associated with prognosis and supports early risk stratification and individualized management.

In summary, IAV can induce refractory AHR, with higher risk in patients with COPD. Early identification of the AHR phenotype and individualized adjustment of respiratory support and pharmacotherapy are essential, with ECMO serving as a rescue therapy when necessary to improve outcomes. Future studies should include larger cohorts with dynamic monitoring of blood gases and inflammatory markers to better define optimal intervention timing and predictors of treatment response.

## Data Availability

The original contributions presented in the study are included in the article/Supplementary Material, further inquiries can be directed to the corresponding author.
